# Consumer Acceptance of Gene-Edited versus Genetically Modified Foods in Korea

**DOI:** 10.3390/ijerph18073805

**Published:** 2021-04-06

**Authors:** Eunae Son, Song Soo Lim

**Affiliations:** Department of Food and Resource Economics, Korea University, Seoul 02841, Korea; 32sea@naver.com

**Keywords:** consumer acceptance, gene-editing, conjoint analysis, genetically modified, soybean oil, Korea

## Abstract

Food made with gene-editing has received considerable attention in recent years because it is claimed to be a little different from traditional genetically modified breeding methods concerning safety. However, consumer acceptance of these novel foods and their potential market uptake remains to be answered. This study aims to assess differences in the acceptance of gene-edited and genetically modified foods in Korea. The choice-based conjoint analysis is adopted to estimate part-worth functions for the soybean oil attributes with 200 surveyed samples. The estimated part-worth values reveal how much each attribute affects consumers’ decision-making. Estimated results suggest that consumers tend to accept gene-editing more than genetically modified foods. The acceptance of novel technology is shown to correspond closely to the degree of consumers’ scientific knowledge, highlighting the importance of revealing relevant information regarding the technology. Results also show that country of origin is a significant food-specific attitudinal factor in shaping consumer preferences.

## 1. Introduction

Consumer perception of genetically modified (GM) crops differs from their scientifically verified safety. Scientists agree that GM crops are safe and have the potential to provide real benefits to humanity. According to the research by the World Health Organization, the American Medical Association, the United States National Academy of Sciences, and the British Royal Society, consuming GM foods is not more dangerous than consuming foods modified by traditional breeding methods [1]. Research has also shown that GM foods are useful for their characteristics, such as resistance to pests and viral diseases and nutrition improvement [1].

However, improving the desired characteristics of food through GM technology is time-consuming and complex, and most importantly, consumers still perceive it as artificial and are concerned about potential risks [2,3]. According to a 2018 survey of 800 Koreans by the Korea Biosafety Clearing House, approximately 50% of the respondents perceived GM foods as a threat to health, and approximately 80% thought that GM foods should be regulated [4].

Meanwhile, a new technology called gene-editing (GE) emerged in 2003. Unlike GM technology, GE does not involve inserting genes from external organisms; rather, to improve various crop characteristics, only the target genes are identified then cut and modified as if with scissors. GE is quickly replacing GM owing to its higher success rate, accuracy, and ease of use [5]. Also referred to as “genetic scissors” and currently the third-generation Clustered Regularly Interspaced Short Palindromic Repeats (CRISPR) technique is mainly being applied and since GE does not involve inserting foreign genes, consumer acceptance is relatively high compared to GM [2,3].

As such, internationally, there is a movement to understand the future direction of GE technology. Based on these trends of GE technology, stakeholders such as farmers, companies, and policymakers must also determine in advance the degree of risk from GE technology that consumers perceive and the impact on future consumer demand, and respond to this proactively. For this purpose, studies on consumer evaluation of GE technology are needed. Particularly, to better grasp the consumer acceptance of GE technology, it must be compared with that of existing GM technology. Incidentally, since soybeans currently have the largest GM crop cultivation area in the world, and GE technology is also used on soybeans in the United States, there is a need for research on consumer evaluation of soybeans. Moreover, even with the same GM and GE technologies, consumer acceptance varies depending on if the technology is applied to food or non-food [6,7]. Therefore, considerations for this are also needed.

Accordingly, this study compares consumer preferences and willingness to pay (WTP) through the choice-based conjoint analysis including both general product attributes and production technology attribute, that is, whether GM and GE technology is applied. The investigation is conducted on a soybean oil as the food and a cotton t-shirt as the non-food, the former uses GM soybeans as raw material and the latter uses GM cotton. While many previous studies have evaluated consumer preferences for GM foods, very few have investigated GE foods [3]. Particularly, this study is differentiated from previous research in that there are no studies are comparing the acceptance of using GM and GE technology on food and non-food [6,7], and will help future stakeholders understand the trends of consumer acceptance of GE technology and respond proactively.

## 2. Literature Review

Table 1 summarizes previous studies on the consumer evaluation of GM and GE foods. The price premium percentage for non-GM foods compared to GM and GE varies with the survey period, items, countries, and methods. Particularly, there are numerous studies on the consumer evaluation of GM foods, including cooking oil, rice, tofu, pork, and beef, and Rousu et al. [8] reported that the acceptance of GM technology on foods subject to extensive processing was higher than fresh foods. Surveyed countries include the United States producing the largest amount of GM foods, Asian countries such as Korea, China, and Japan, and European countries mainly importing GM foods. Regarding survey methods, the contingent valuation method (CVM), the conjoint analysis, and the experimental auction have been used [6,7,8,9,10,11,12,13,14,15,16,17,18,19,20].

Conversely, there is very little research on the consumer evaluation of GE foods; particularly, a soybean oil, the survey item examined in this study, has not been surveyed. Shew et al. [3], the only consumer evaluation study on GE technology, compared consumers’ willingness to consume (WTC) for GE and GM rice in the United States, Canada, Belgium, France, and Australia. As a result, the WTC for GE rice was higher than GM rice and the usefulness and safety of GE rice were also higher. That is, consumers were accepting GE technology as a different one from GM. In a similar vein, Shew et al. [2] compared the WTC and WTP for rice grown with RNA-interference which is a biological mechanism used to selectively silence the expression of a specific gene and GM rice in the United States, Canada, Belgium, France, and Australia. It showed that WTC and WTP were higher for rice grown with RNA-interference than GM rice.

Besides, few studies have compared consumer evaluations of GM or GE foods with non-foods. A related study, Christoph et al. [6] compared the consumer preferences for GM French fries made and GM potato paper and reported that the WTP for GM French fries was lower than the other. The recent study of Berning and Campbell [7] also surveyed consumer preferences of tomatoes, tomato plants, geranium (ornamental plant), and turf with “no labels”, “not-certified GMO-free”, and “certified GMO-free”. The consumers’ utility of “certified GMO-free” was high in the order of tomatoes, tomato plants, geranium, and turf. These two studies suggest that consumers were more sensitive to the safety of GM foods than non-foods.

Meanwhile, among the survey methods, the CVM [23] and the conjoint analysis [24] have a common aspect in that assume hypothetical conditions. However, the conjoint analysis differs in that it composes the hypothetical choice options including the product’s attributes and then asks consumers for their preferences, thus indirectly analyzing the WTP by estimating the trade-off between multiple attributes. Thus, while there is still a hypothetical bias, it is relatively similar to actual purchase conditions, so it is the most frequently applied to consumer evaluation. Particularly, the choice-based conjoint analysis choosing the best one among the choice options is widely used than the conventional conjoint analysis evaluating the choice options by ranking or scoring.

Meanwhile, the experimental auction method [25], more similar to actual purchase conditions, can derive more accurate WTP than CVM and the conjoint analysis because it gives real money to respondents to induce their purchase choices. However, it is very costly and time-consuming and inapplicable to this study since GE foods and non-foods have not yet been commercialized; as such, this study applied the choice-based conjoint analysis, which can most realistically induce the consumer’s WTP in a given environment.

To sum up, the previous studies are mainly concerned with the consumer acceptance of GM technology. However, this study investigates the consumer acceptance of GE technology and compares this with GM and is further differentiated from previous researches in that the targets of technology application are classified into food and non-food products.

## 3. Methods

Among various conjoint analysis methods, this study applied the choice-based conjoint analysis. The choice-based conjoint analysis is based on two key economic theories: Lancaster’s consumer theory [26] and McFadden’s random utility theory [27]. The former states that a product’s value can be expressed as a sum of the utility for each attribute because the consumer’s utility is derived not from the product itself but the product’s attributes. If product j has m attributes and *n* attribute levels, then the utility $U_{ij}$ that respondent i feels toward product j can be expressed as the sum of each utility *β* for *n* attribute level variables *Z*, where *β* is the part-worth for each attribute level. Equation (1) is the part-worth function model:

(1) $$U_{ij}=\beta_{1}Z_{i11}+\beta_{2}Z_{i21}+\cdots +\beta_{n}Z_{imn}+\epsilon_{ij}$$

McFadden [27] stated that consumer utility is maximized and manifests as consumer behavior in the market and that consumer utility also includes a stochastic part that cannot be measured (e.g., individual consumer perceptions or attitudes). This is the random utility theory, which states that it is difficult to completely determine all the features that influence the consumer’s purchasing decision process. Therefore, the consumer’s utility is divided into two parts [28]. In Equation (2), $V_{ij}$ is the observable part of consumer utility, such as product attributes, and $\epsilon_{ij}$ is the unobservable part, such as individual unique characteristics:

(2) $$U_{ij}=V_{ij}+\epsilon_{ij}$$

Consumers select the highest-utility product among various products; since it is difficult to perfectly know the consumer’s utility containing a stochastic part, the consumer choice is expressed as a probability. That is, since respondent *i* will select alternative (product) *j* if the utility obtained from alternative j is higher than alternative *k*, the probability that respondent *i* will select alternative *j* can be expressed as Equation (3).

(3) $$P_{ij}=Pr\left(U_{ij}>U_{ik}\right)=Pr\left(V_{ij}+\epsilon_{ij}>V_{ik}+\epsilon_{ik}\right)=Pr\left(V_{ij}-V_{ik}>\epsilon_{ik}-\epsilon_{ij}\right),\;\;\;\forall \;j\neq k$$

The cumulative distribution probability in Equation (3) can be expressed as in Equation (4) using the joint probability density function $f\left(\epsilon_{i}^{\prime }\right)$ of the error term vector $\epsilon_{i}^{\prime }=\left(\epsilon_{i1},\ldots ,\epsilon_{iJ}\right)$. $I_{ij}$ is an indicator function that is 1 when respondent *i* selects alternative j and 0 if otherwise, and $P_{ij}$ is classified into a logit model when the joint probability density function *f*($\epsilon_{i}^{\prime })$ of the error term vector $\epsilon_{i}^{\prime }$ is assumed as logistic distribution and a probit model when assumed as a standard normal distribution [29]:

(4) $$P_{ij}=\int_{\epsilon }I_{ij}\left(V_{ij}-V_{ik}>\epsilon_{ik}-\epsilon_{ij}\right)f\left(\epsilon_{i}^{\prime }\right)d\epsilon_{i}^{\prime }$$

In this study, $f\left(\epsilon_{i}^{\prime }\right)$ was assumed as a logistic distribution; generally, in the choice-based conjoint analysis, the logit model is applied more than the probit model because of its computational advantages [30]. To achieve a more realistic investigation, this study used a multinomial logit model including a “no choice” alternative, besides the two alternatives consisting of different attribute levels. That is, to reduce bias in the investigation, if neither alternatives maximize the consumer’s utility, then the selection is delayed to find a better alternative [31].

The multinomial logit model assumes independence from irrelevant alternatives (IIA). In other words, the ratio of the probability of selecting alternative A to the probability of selecting alternative B is not influenced by the existence of alternative C [32]. Though this is a rather strong assumption, in reality, it is advantageous to secure the statistical significance of the coefficient values when it is difficult to determine the correlation between the alternatives [33].

Furthermore, the multinomial logit model assumes that the error term is independently and identically distributed and a Gumbel distribution, which is similar to a normal distribution, its tails are flatter, making it suitable for extreme data [34]. Based on this assumption, the probability that respondent *i* will select alternative *j* in Equation (4) can be simply expressed as Equation (5) [35]:

(5) $$P_{ij}=\frac{\exp\left(V_{ij}\right)}{\sum_{i=1}^{I}\sum_{j=1}^{J}\exp\left(V_{ij}\right)}$$

The log-likelihood function used to find the Maximum Likelihood Estimator (MLE) of $P_{ij}$ can be expressed as in Equation (6); as respondent *i*’s selection of alternative *j* becomes “Yes” or “No”, the indicator function $I_{ij}$ is included:

(6) $$lnL=\overset{I}{\underset{i=1}{\sum }}\overset{J}{\underset{j=1}{\sum }}I_{ij}\cdot lnP_{ij}$$

When applying the MLE method to the log-likelihood function of Equation (6), the values of the necessary parameters are estimated. The empirical model in this study expresses the observable part of consumer utility $V_{ij}$ and $V_{ij}^{\prime }$ as attribute variables including price, as shown in Equations (7) and (8). In Equation (7), price is recognized as a continuous variable and the origin of raw material and the production technology as categorical variables and are treated as dummy variables for each level. In Equation (8), all variables are recognized as categorical variables:

(7) $$V_{ij}=\beta_{1}Z_{i11}+\beta_{2}Z_{i12}+\beta_{3}Z_{i21}+\beta_{4}Z_{i22}+\beta_{5}Z_{i3}$$

(8) $$V_{ij}^{\prime }=\gamma_{1}Z_{i11}+\gamma_{2}Z_{i12}+\gamma_{3}Z_{i21}+\gamma_{4}Z_{i22}+\gamma_{5}Z_{i31}+\gamma_{6}Z_{i32}+\gamma_{7}Z_{i33}+\gamma_{8}Z_{i34}$$

Among the origin of the raw material, $Z_{i11}$ represents domestic and $Z_{i12}$ is US; regarding the production technology, $Z_{i21}$ represents GM and $Z_{i22}$ is GE; $Z_{i3}$ is the price recognized as a continuous variable, and among the prices recognized as a categorical variable, $Z_{i31}$ is $2.9 for a soybean, $6.3 for a cotton t-shirt, $Z_{i32}$ is $3.3, $8.4, $Z_{i33}$ is $3.7, $10.5, and $Z_{i34}$ is $4.1, $12.6. The reference of the origin of raw material is China, that of the production technology is non-GM, and that of the price is $2.5, $4.2. *β* and *γ* are coefficients for individual attribute levels that affect the respondent’s utility and parameters to be estimated. After substituting each of Equations (7) and (8) into Equation (5), substituting these into Equation (6), and then applying MLE, an estimate for *β* and *γ* can be obtained.

Additionally, by totally differentiating Equation (7), the marginal willingness to pay (MWTP) for each attribute level can be obtained as in Equation (9). This can be regarded as the marginal rate of substitution between the income and an individual attribute level, based on the estimated coefficient for the price is the same as the marginal utility of money [36]:

(9) $$MTWP_{z_{imn}}=\frac{dV/dZ_{imn}}{dV/dZ_{i3}}=-\frac{\beta_{n}}{\beta_{5}}$$

Furthermore, based on the estimation coefficient in Equation (8), each attribute’s relative importance can be obtained. The relative importance is calculated in a manner described by Halbrendt et al. [37] (Equation (10)). First, the highest and the lowest part-worth utilities (coefficients) are determined for each attribute and the difference between two establishes the attribute’s utility range. Then, the relative importance of the sth attribute is calculated as follows:

(10) $$RI_{z_{is}}=\frac{Utility\;Range_{z_{is}}}{\sum_{i=1}^{I}\sum_{m=1}^{M}Utility\;Range_{Z_{im}}}\times 100$$

## 4. Materials

### 4.1. Survey Overview

The survey was divided into a preliminary survey and the main survey; the preliminary survey was conducted online from 1 July to 3 July 2019, with 44 adults in their twenties to sixties, through which the questions’ composition was checked and ambiguous phrases were modified. Finally, 39 questions were determined (the response time of 7 min). The main survey was conducted on 11 July 2019 through “Open Survey”, a professional survey agency, with 200 adults on a mobile application. The sample of the main survey was randomly stratified into partitions of 50% males and females and 25% for each age group from the twenties to fifties, and the confidence level of the sample was 95%. Regarding a final education level, 84% of the sample were university graduates or higher, and the average annual household income was approximately $45,975 (Table 2).

The survey largely comprised two parts. The first consisted of the questions about the respondents’ demographic information (gender, age, final education level, average annual household income, children’s age, etc.) and the respondents’ perception of the GM and GE technologies, including awareness of two technologies, a scientific knowledge level, awareness of the technology safety difference between the two technologies, and sensitivity to food safety. The second part was the choice-based conjoint analysis of a soybean oil, food and a cotton t-shirt, non-food. Alternatives consisting of different attribute levels and prices for each product were presented to the respondents, and the respondents selected their most preferred alternative. Through this, the part-worth and WTP concerning the changes in the attribute levels were estimated. The attributes used in the choice-based conjoint analysis were the raw materials’ origin, the production technology, and the product price; the raw materials’ origin was divided into Korea, US, and China, and the production technology was divided into non-GM, GM, and GE. The product price of a soybean oil was classified into five prices, $2.5, $2.9, $3.3, $3.7, and $4.1 using the difference between the average market price, $3.3, and both end prices as 25% of the average market price for 900 mL of a soybean oil. And, since a cotton t-shirt has a higher average market price than a soybean oil, a cotton t-shirt’s price band was divided into $4.2, $6.3, $8.4, $10.5, and $12.6 using the interval between the average market price, $8.4, and both end prices as 50% of the average market price for U crew necks of a specific Specialty store retailer of Private label Apparel (SPA) brand (Table 3).

For the hypothetical choice sets, to reduce the load placed on consumers, SPSS orthogonal design was used to derive the minimum choice sets and the unreasonable choice sets were excluded, thus finally obtaining 10 choice sets for each soybean oil and cotton t-shirts. The choice set pictures are presented as shown in Table 4, and the respondents can select their preferred hypothetical product between alternative 1 and alternative 2. If they prefer neither alternative, then they can select alternative 3, “no choice”.

### 4.2. General Characteristics of the Respondents

Approximately 85% of the respondents responded that they had heard of GM, whereas less than half, at less than 45%, were aware of GE (Table 5). This indicates that since GE technology has emerged more recently than GM technology and has not yet been commercialized in Korea, the interest of respondents in it has been relatively low.

The respondents’ scientific knowledge level of GM technology was evaluated through two questions; the first was a relatively low-difficulty question, and the second question was high in difficulty-level. The correct answer rate for the first question was 41%, and that for the second was 29%, lower than the first. The fact that both rates were less than 50% indicates that the scientific knowledge level of GM technology is generally low. However, given the 29.6% correct answer rate for questions similar to the first question in Han et al. [38], it is evident that false perceptions of GM technology have declined compared to a decade ago.

Next, regarding the difference in technology safety perceived by respondents for GM and GE technology, 51% of the respondents, the largest group, responded that GE technology is safer, whereas 7%, the smallest group, responded that GM technology was safer than GE technology. This indicates that the respondents think that GE technology is relatively safer than GM technology.

Furthermore, to evaluate sensitivity to food safety, the respondents were asked if they tend to purchase food made from organic raw material, to which 59% responded in the affirmative and 41% in the negative. Thus, the proportion of consumers sensitive to food safety was about 20% higher.

Meanwhile, to confirm the awareness of GM labeling, the respondents were asked whether there is a soybean oil made from domestic non-GM soybeans among the large domestic food brands. Surprisingly, to which 48%, the largest group, responded “I do not know.”, 35% responded “Yes,” and only 18% responded “No.” In fact, since the soybean production in Korea is very small, there is no soybean oil made from domestic soybeans, but that made from imported GM soybeans among the large domestic brands. That is, consumers were not correctly aware of the origin of soybeans from a soybean oil on the domestic market. This is because a soybean oil is not required GM labeling, thereby consumers inevitably purchase it without accurate information about the raw material. According to the “Labeling Standards for Genetically Modified Foods, etc.”, regarding the product in which no GM genes remain due to a refining to a high degree, it is exempted from GM labeling [39]. Accordingly, to protect consumers’ rights to know, consumer civic groups are demanding “Complete Labeling” requiring a GM label if any GM raw material are used, regardless of whether GM genes are detected in the final product.

## 5. Results

### 5.1. Part-Worth by an Attribute Level

An estimate for *β* and *γ* can be calculated by applying MLE to the log-likelihood function in Equation (6), which signifies the part-worth for each attribute level that affects the respondent’s utility. Since this was estimated by the logit model, the estimated coefficient value for each attribute level means the influence of each attribute level on the probability of selecting an alternative, the dependent variable. The Wald test validating the null hypothesis of the estimated coefficient values was performed to examine the model’s goodness-of-fit [40], confirming that all estimated coefficient values were statistically significant (Table 6). The coefficient values of Models 1 and 2 were similar; regarding the origin of raw material, both coefficients of a soybean oil and a cotton t-shirt were statistically significant positive numbers, showing alternative selection probabilities of domestic origin and US-origin were higher than China-origin because of the former’s higher part-worth. Particularly, the alternative selection probability for domestic origin was higher than US-origin.

Regarding the production technology, both coefficients of soybean oil and a cotton t-shirt were statistically significant negative numbers, indicating alternative selecting probabilities of GM and GE were lower than non-GM because of the former’s lower part-worth. For a soybean oil, however, the alternative selection probability for GM was lower than GE, whereas, for a cotton t-shirt, that for GE was vice versa. Additionally, the coefficient of the price was a statistically significant negative number, which supports the general demand theory that the probability of selecting an alternative decline as the price rises.

### 5.2. Relative Importance by Attribute

From the above estimation results, the relative importance by attribute can be calculated based on the part-worth of Model 2 in Table 6. The relative importance of individual attributes can be estimated as the change in part-worth by an attribute level within an attribute, i.e., the difference between the maximum and minimum values of the part-worth of an attribute level [41] (Table 7). A large difference between the maximum and minimum values of the part-worth of an attribute level signifies that the utility greatly varies with a change in the attribute level and that consumers respond sensitively to the attribute, making it important [41].

According to this study’s relative importance results by attribute, the relative importance of the production technology for both a soybean oil and cotton t-shirts was lower than that of the origin of raw material. Particularly, the relative importance of the production technology for a soybean oil (37%) was lower than that of the origin of raw material (46%), and the difference between the two attributes was large. However, though the relative importance of the production technology for cotton t-shirts (31%) was lower than that of the origin of raw material (34%), the difference between the two attributes was small. Hence, when purchasing food products rather than non-food products, the respondents regarded the origin of raw material as more important than the production technology.

Moreover, the relative importance of the product price for cotton t-shirts was 35%, higher than the relative importance of the production technology and the origin of raw material at 31% and 34%, respectively. In contrast, the relative importance of the product price for soybean oil was 17%, lower than the relative importance of the production technology and the origin of raw material, 37% and 46%, and also lower than the relative importance of the product price for cotton t-shirts. Hence, when purchasing cotton t-shirts, a non-food product, as opposed to a soybean oil, a food product, the respondents consider price more than quality-related attributes. Conversely, when purchasing a soybean oil, food, if the presented price is within the range of the market price, the respondents prioritize other attributes considered more important than price [41,42]. Thus, the respondents are less sensitive to the price attribute when purchasing foods such as a soybean oil than non-foods, such as cotton t-shirts.

Additionally, comparing the total sum for the part-worth range of each attribute of a soybean oil and cotton t-shirts, a soybean oil was 2.82 and cotton t-shirts was 2.78, indicating that the change in an alternative selection probability, according to an attribute level, is greater for a soybean oil than cotton t-shirts. Hence, when given the production technology, the origin of raw material, and the product price as product attributes, the respondents are more sensitive to changes in these attributes and show higher involvement when purchasing a soybean oil, food, than cotton t-shirts, non-food.

### 5.3. Marginal Willingness to Pay by an Attribute Level

Using the above estimation results in Table 6, the MWTP by an attribute level can also be calculated based on Model 1. The MWTP by an attribute level was estimated using the Wald test (Table 8). The Wald test uses the Wald distance or the difference between the estimated coefficient value and the coefficient value of the null hypothesis; the null hypothesis is rejected if the difference is statistically large. 

In this estimation, the null hypothesis is as presented in the above model:

$$-\frac{Part-worth\;estimated\;value\;of\;the\;specific\;attribute\left(\beta_{n}\right)}{Part-worth\;estimated\;value\;of\;price\left(\beta_{p}\right)}=0$$

According to the Wald test results, the MWTPs by an attribute level of a soybean oil and a cotton t-shirt were significantly at almost 1% level.

Regarding the production technology of a soybean oil, as shown in the part-worth results presented above, the penalty for GE compared to non-GM was 6162 won, lower than the penalty for GM of 7557 won. Hence, the respondents exhibited a higher acceptance of GE technology, which does not involve inserting foreign genes, compared to GM technology, which inserts foreign genes. However, for a cotton t-shirt, the penalty for GE compared to non-GM was 16,025 won, higher than the penalty for GM of 10,220 won. This result, as shown by the relative importance by an attribute, is because the respondents base their decision primarily on the product price rather than the production technology when selecting an alternative for a cotton t-shirt. The choice-based conjoint analysis for a cotton t-shirt is structured so that, if lower prices are prioritized when selecting a choice option, then between GE and GM, the rate at which GM is selected is about 33%, higher than the rate at which GE is selected (11%).

Moreover, in the case of the origin of raw material, the MWTPs of a soybean oil and a cotton t-shirt for domestic raw materials, compared to Chinese raw materials, were 8315 won and 17,027 won, respectively, both of which exceeded the MWTPs for US raw materials compared to Chinese raw materials of 2168 won and 4956 won. Thus, the respondents preferred domestic raw materials to Chinese and US (i.e., imported) raw materials. Meanwhile, the MWTPs for domestic raw materials, compared to Chinese raw materials, were higher than the penalty for GM or GE compared to non-GM; It can be seen that, though the respondents prefer non-GM raw materials to GM or GE, their preferences for domestic raw materials over Chinese raw materials is higher. Therefore, consumers have more confidence in food safety in terms of the origin of raw material than the production technology. Particularly, Korea has the high loyalty toward domestic products due to the concept of “Sintoburi”, which means that food produced from the land where one were born suits one’s body the best, so it tends to show strong preferences for domestic products to imported products [43].

**Figure 1:**
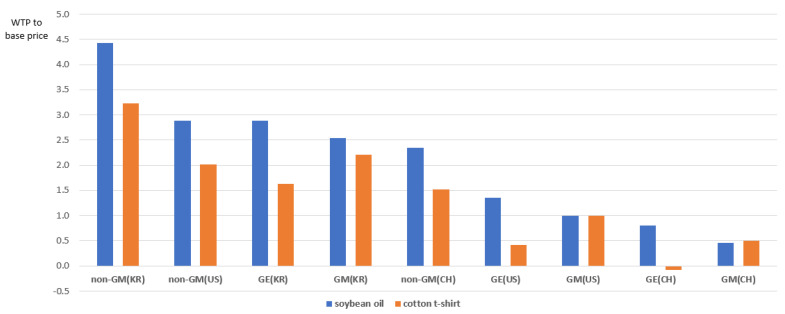
WTP to the base price by an attribute level combination.

The above attribute levels were combined to form hypothetical products, and the WTPs compared to the base prices were calculated (Figure 1). The base prices are the market prices set up in a survey design ($3.3 for a soybean oil and $8.4 for a cotton t-shirt), which are the average prices of products with US GM raw materials and currently distributed on domestic markets. For both a soybean oil and a cotton t-shirt, the domestic non-GM choice option had the highest preference, and as for the WTPs compared to the base prices, a soybean oil was 4.4 and a cotton t-shirt was 3.2, with the former showing a higher value. In other choice options also, the WTP to the base price for a soybean oil was higher overall, indicating that the respondents are more sensitive to the production technology and the origin of raw material when purchasing food products, such as a soybean oil, than non-food products, such as a cotton t-shirt, as mentioned above. Christoph et al. [6] and Berning and Campbell [7] also compared the sensitivity to GM technology between foods and non-foods and reported that the sensitivity to GM technology for non-foods was relatively low.

Table 9 compares the WTP obtained through the choice-based conjoint analysis with the WTP obtained through the CVM and the actual market product price for the product with domestic non-GM raw material, derived as the most preferred option above.

First, the average WTPs obtained through the CVM for the products with domestic non-GM raw material were 5500 won for a soybean oil and 14,500 won for a cotton t-shirt, approximately 1.5 times the base prices, 4000 won and 10,000 won. These were lower than the WTPs for a soybean oil (17,704 won = 4000 won (base price) + 6147 won (MWTP for domestic raw material compared to US raw material) + 7557 won (MWTP for non-GM raw material compared to GM raw material), 4.4 times the base price) and cotton t-shirts (32,291 won = 10,000 won (base price) + 12,071 won (MWTP for domestic raw material compared to US raw material) + 10,220 won (MWTP for non-GM raw material compared to GM raw material), 3.2 times the base price) investigated via the choice-based conjoint analysis. This result seems to be because of the difference in the WTP derivation method. The CVM is relatively unrealistic because it asks respondents’ WTPs for one virtual product rather than comparing multiple virtual products and responding their WTPs. However, since the choice-based conjoint analysis asks respondents for their preferences among the several choice options comprising numerous attributes, it can derive a more realistic WTP.

Second, the WTPs for a domestic non-GM soybean oil and cotton t-shirt investigated via the choice-based conjoint analysis were compared with several market prices. If the raw materials are domestic, the WTPs for these, 17,704 won for a soybean oil and 32,291 won for a cotton t-shirt, are higher than the market prices of imported organic products, 11,000 won for a soybean oil and 20,000 won for a cotton t-shirt, though the domestic raw materials are not organic. The market price of a domestic non-GM soybean oil (32,000 won), eight times the base price, was higher than the consumer’s WTP investigated via the choice-based conjoint analysis (17,704 won). This seems to be there are few domestic soybean productions, and among them, there are few used for processing a soybean oil, so the supply cost burden was reflected in the actual price. Owing to this high cost, among the soybean oil currently sold by domestic large food brand companies, none is made from domestic soybeans; such a soybean oil is only sold in small quantities in small farms.

### 5.4. MWTP Variation Range for the Production Technology by Respondents’ Characteristics

This study compared the variation ranges in the MWTPs for the production technology of a soybean oil and a cotton t-shirt according to the respondents’ characteristics. A total of four main characteristics were examined: a scientific knowledge level, sensitivity to food safety, sensitivity to technical safety, and a children’s age. The variation range means the difference between the maximum and minimum MWTP values according to the characteristics of the respondent (Table 10). As a result, in general, the variation range in MWTP according to sensitivity to technical safety was the largest, followed by a children’s age, a scientific knowledge level, and sensitivity to food safety. Unexpectedly, overall, the MWTP greatly varied according to the respondents’ scientific knowledge level compared to their sensitivity to food safety. 

Sensitivity to technical safety showed a larger change in MWTP than a children’s age. This indicates that, regardless of the differences of food safety sensitivity and a children’s age, if the respondents’ scientific knowledge is improved and technology safety sensitivity is lowered accordingly, the penalty for GM or GE raw materials compared to non-GM raw material may be lowered. This is similar to the findings of previous studies [2,3]; in the consumer survey, when more information was provided to respondents on GE technology than simple information, the respondents’ sensitivity to GE rice decreased, and regarding respondents’ education level, the university graduate respondents were less sensitive to rice grown using New Plant Breeding Techniques (NPBTs) than high school graduates or below.

## 6. Discussion

This study is differentiated from previous researches in that whereas the prior studies mainly investigated the consumer acceptance of GM technology, this study investigated the consumer acceptance of GE technology and compared this with that of GM technology; moreover, this study classified the targets of technology application to food and non-food products and analyzed the difference in consumer acceptance between the two.

The following implications can be derived based on the estimation results: As shown in the estimation results of part-worth and MWTP by an attribute level, regarding the production technology, consumers did not prefer GM and GE raw materials over non-GM raw materials when purchasing a soybean oil and a cotton t-shirt. And when purchasing a soybean oil, the penalty for GM raw material was higher than that for GE. This suggests that consumers will show less resistance to future food products that use GE technology than GM technology.

Meanwhile, regarding the relative importance, when purchasing foods as opposed to non-foods, respondents focused on quality-related attributes rather than price in their selections and reacted less sensitively to price. And, for both products, the relative importance of the origin of raw material was higher than the production technology. This is similar to the findings of Stanton et al. [41] and Yang [44], in which the preference for the origin of raw materials was greater than that for the production method (e.g., whether or not the raw material is organic) when purchasing apples and tofu, implying that the respondents are more confident in food safety represented by the raw material’s origin than by the production method.

Also, among the origin of raw material, the consumer preference for domestic raw material was especially high, reflecting the strong influence of “Sintoburi,” a Korean belief that agricultural products grown in their land of birth are better suited to their body. This has similarities to “Local Production” in Japan, the “Slow Food Movement” in Italy, and the “Local Food Movement” in the US and Europe. However, while these trends are alternatives for food safety and the sustainability of agriculture, the local community, and the ecological environment, “Sintoburi” includes high loyalty for domestic brands, i.e., native species, in addition to this [43]. This tendency also applies to the concept of “Geographical Indication” (GI) for local agricultural products in Korea, in which location names are granted trademark rights to protect local agricultural products with unique characteristics [45].

Additionally, consumers respond more sensitively to GM and GE technology based on their scientific knowledge level than their food safety sensitivity, and technology safety sensitivity rather than a children’s age. Therefore, if respondents are highly sensitive to food safety and have young children, but their scientific knowledge about GM and GE technology is enhanced sufficiently, their anxiety toward the technologies can be greatly reduced. Accordingly, when introducing GE technology to consumers, the objective information about the technology must be provided enough at the government and private level. Particularly, concerning the technology safety, a process of education and promotion about the differences between GE and GM technology is also necessary, which should be implemented more actively when the technologies are applied to food.

The limitations of the study are mainly the characteristics of design focusing on consumer acceptance, choice and attitudes for GM and GE technology. Discussions about potential risks of new techniques, scientific risk assessment or environmental monitoring for novel products go beyond the scope of this study. Meanwhile, to derive more realistic results for consumers’ WTP, the investigation must be performed under conditions similar to reality. However, since GE foods have not yet been commercialized, the investigation was conducted under hypothetical conditions, thereby introducing the limitation of the hypothetical bias in the consumers’ WTP. Additionally, to improve the respondents’ understanding of GM and GE technology, they must be directly provided information face-to-face; however, owing to time and cost limitations, they were provided information through non-face-to-face methods instead. Therefore, once GE foods are commercialized in the future, it is necessary to investigate consumers’ WTP under conditions similar to reality (e.g., the experimental auction) and provide information on GE and GM technology face-to-face.

Furthermore, while this study investigated consumers’ WTP regarding general GE technology, in fact, their WTP can vary among the different benefits of GE technology, such as herbicide resistance, pest resistance, and nutrition improvement [46]. Accordingly, a comparative analysis of these is needed and it can help improve understanding of specific GE technology with low consumer resistance. And, a research comparing Korean consumers’ acceptance of GE technology with other countries will be able to identify the degree of Korean consumer acceptance in the world. Additionally, just as consumer acceptance of applying GM technology to processed foods is relatively high compared to that of foods originally perceived as natural (e.g., fruits or nuts) [47], by examining the GE technology acceptance by different items even in the same food category, it will be possible to identify the food items for which GE technology is prioritized.

## 7. Conclusions

The choice-based conjoint analysis shows that the consumer penalty for gene-edited raw materials is actually lower than that for genetically-modified ones in the case of soybean oil. This sheds light on bridging the gap between science and consumers on the new technology. The findings that consumer acceptance of gene-edited technology is closely linked and corresponds to the degree of consumers’ scientific knowledge suggest the importance of appropriate risk communication and a wide spread of scientific information in the public and private domain.

Estimated results also show that country of origin is a significant food-specific attitudinal factor in shaping consumer preference. Although country of origin is not a food safety issue, the vast majority of Korean consumers have confidence in the safety of locally grown produce and food. Since the country has not approved any genetically-modified crops for commercial production, the prevailing Korean belief in “Sintoburi” is recognized as a statistically significant determinant of consumer preference.

## Figures and Tables

**Table 1:** Summary of the previous studies on the consumer evaluation of GM and GE foods.

Compared to	Study	Product	Country	Valuation Method	Price Premium for Non-GM (%)	
GM ^2^	Buhr, Hayes, Shogren, and Kliebenstein [9]	Pork sandwich	US	Auction	−15.44	
	Boccaletti and Moro [10]	Food	Italy	CVM ^1^	1.06	
	Baker and Burnham [11]	Cornflakes	US	Conjoint (ranking)	39.84	
	Chern, Rickertsen, Tsuboi, and Fu [12]	Vegetable oil	US Norway Japan Taiwan	Conjoint (choice)	56.00 62.00 36.50 19.00	
	Kwon [13]	Tofu	Korea	CVM	44.7~136	
	Kwon and Kim [14]	Chocolate bar	Korea	Auction	104.00 (student) 356.00 (housewife)	
	Li, Curtis, McCluskey, and Wahl [15]	Rice Soybean oil	China	CVM	−38.00 −16.30	
	Lusk [16]	Golden rice	US	CVM	−19.54	
	Lusk and Fox [17]	Beefsteak	US UK Germany France	Conjoint (choice)	38.94 74.24 90.24 109.65	
	Rousu, Hffian, Shogren, and Tegene [8]	Vegetable oil Corn chips Potato	US	Auction	5.26 10.29 12.00	
	Lusk, House, Valli, Jaeger, Moore, Morrow, and Traill [21]	Cookie	TX, US CA, US FL, US England France	Auction	40.00 80.00 20.00 160.00 784.00	
	Christoph, Roosen, and Bruhn [6]	French fries Potato paper	Germany	Conjoint (choice)	106.32 ^3^ 91.23 ^4^ −83.22 ^3^ −1.00 ^4^	
	Jin [18]	Rice	China	CVM	89.00	
	Berning and Campbell [7]	Fresh tomato Tomato plants Geraniums Turf	US	Conjoint (ranking)	-	
	Delmond, McCluskey, Yormirzoev, and Rogova [19]	Bread	Russia	Conjoint (choice)	197.00	
	Martinez-Ribaya and Areal [20]	Soya-based product	Argentina	CVM	50.00	
RNAi ^5^, GM	Shew, Danforth, Nalley, Nayga, Tsiboe, and Dixon [2]	Rice	US Canada Belgium France Australia	Field experiment	RNAi	GM
					152.40 87.60 - 98.40 84.80	251.20 179.40 173.20 267.00 159.00
GE ^6^, GM	Shew, Nalley, Snell, Nayga, and Dixon [3]	Rice	US Canada Belgium France Australia	Field experiment	GE	GM
					91.60 23.40 31.80 42.40 44.80	96.00 18.40 32.00 42.00 44.20

Note: Compilated by authors with reference to Lusk, Jamal, Kurlander, Roucan, and Taulman [22]. ^1^ Contingent Valuation Method. ^2^ Genetically Modified. ^3^ For the reduction of spreading risk. ^4^ For the reduction of pesticide. ^5^ RiboNucleic Acid-interference. ^6^ Gene-Edited.

**Table 2:** Summary statistics.

Description		*N*	%
Gender	Male	100	50
	Female	100	50
Age	20 to 29	50	25
	30 to 39	50	25
	40 to 49	50	25
	50 to 59	50	25
Final education level	High school graduation	31	16
	University attending or graduation	155	77
	Graduate school or higher	14	7

**Table 3:** An attribute and an attribute level.

Attribute		Attribute Level
Origin of raw material		Korea
		US
		China
Production technology		Non-GM
		GM
		GE
Product price	Soybean oil (900 mL)	$2.5 (3000 won), $2.9 (3500 won), $3.3 (4000 won), $3.7 (4500 won), $4.1 (5000 won)
	Cotton t-shirt (a specific SPA brand)	$4.2 (5000 won), $6.3 (7500 won), $8.4 (10,000 won), $10.5 (12,500 won), $12.6 (15,000 won)

**Table 4:** The example of the presentation format of a choice set.

Alternative 1	Alternative 2	Alternative 3
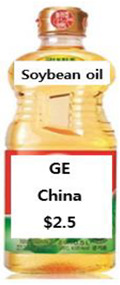	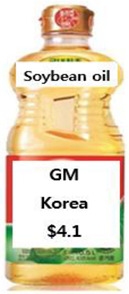	No choice
(√)	( )	( )

**Table 5:** Survey results of respondents’ general characteristics.

Awareness	Yes		No	
Have you ever heard of GM technology?	169 persons (85%)		31 persons (15%)	
Have you ever heard of GE technology?	90 persons (45%)		110 persons (55%)	
**Scientific Knowledge Level**	**Yes**	**No**	**Correct Answer**	
Can a person’s genes change by eating GM soybeans?	99 persons (59%)	70 persons (41%)	No	
Are GM genes (DNA) left in a soybean oil made by heat-treating GM soybeans?	120 persons (71%)	49 persons (29%)	No	
**Technology Safety**	**Both Are Safe**	**GE Is Safer**	**GM Is Safer**	**Both Are Unsafe**
What do you think about the safety of GM and GE technology?	26 persons (13%)	103 persons (51%)	14 persons (7%)	57 persons (29%)
**Sensitivity to Food Safety**	**Very Much So**	**Somewhat**	**Hardly**	**Not at all**
Do you mainly buy foods made from organic/eco-friendly raw materials?	21 persons (11%)	97 persons (48%)	75 persons (37%)	7 persons (4%)
**Other**	**Yes**	**No**	**I Do Not Know**	**Correct Answer**
Among the domestic large food brands, is there a soybean oil made from domestic non-GM soybeans?	69 persons (35%)	36 persons (18%)	95 persons (48%)	No

**Table 6:** Part-worth by an attribute level.

Attribute	Level	Part-Worth		Model 1		Model 2		Reference
				Soybean Oil	Cotton t-Shirt	Soybean Oil	Cotton t-Shirt	
Origin of raw material	Korea	$\beta_{1}$	$\gamma_{1}$	1.4719 *** (0.1139)	0.9773 *** (0.1040)	1.2924 *** (0.0966)	0.9347 *** (0.0955)	China
	US	$\beta_{2}$	$\gamma_{2}$	0.3838 *** (0.1005)	0.2844 *** (0.0862)	0.4090 *** (0.1162)	−0.0965 (0.0758)	
Production technology	GM	$\beta_{3}$	$\gamma_{3}$	−1.3376 *** (0.0955)	−0.5866 *** (0.0872)	−1.0684 *** (0.0882)	−0.1620 * (0.0899)	non-GM
	GE	$\beta_{4}$	$\gamma_{4}$	−1.0907 *** (0.0898)	−0.9198 *** (0.0829)	−0.8999 *** (0.0952)	−0.8715 *** (0.0857)	
Product price		$\beta_{5}$	$\gamma_{5}$	−0.00017 *** (0.00002)	−0.00005 *** (0.000007)	−0.0958 (0.1029)	−0.1123 (0.0756)	$2.5 $4.2
			$\gamma_{6}$			−0.3090 *** (0.1051)	−0.4168 *** (0.1128)	
			$\gamma_{7}$			−0.3538 *** (0.0778)	−0.7606 *** (0.1057)	
			$\gamma_{8}$			−0.4722 *** (0.0799)	−0.9849 *** (0.1075)	
Log-likelihood				−3753.911	−3934.698	−3848.223	−3937.283	
Wald test (*p*-value)				636.424 *** (0.0000)	386.367 *** (0.0000)	501.9547 *** (0.0000)	376.8171 *** (0.0000)	

Note: * and *** indicate 10%, 5%, and 1% significance levels, respectively and values in parenthesis are standard errors. Also, Model 1 treats the price variable as a continuous variable, and Model 2 treats those as dummy variables based on the lowest price ($2.5, $4.2).

**Table 7:** A relative importance by an attribute.

Attribute	Soybean Oil		Cotton t-Shirt	
	Range	Importance (%)	Range	Importance (%)
Origin of raw material	1.29	46	0.93	34
Production technology	1.06	37	0.87	31
Product price	0.47	17	0.98	35
Total	2.82	100	2.78	100

**Table 8:** MWTP by an attribute level.

Attribute	Level	MWTP (Won)		Reference
		Soybean Oil (900 mL)	Cotton t-Shirt (A Specific SPA Brand)	
Origin of raw material	Korea	8315 *** (807)	17,027 *** (1777)	China
	US	2168 ** (419)	4956 *** (1231)	
Production technology	GM	−7557 *** (1163)	−10,220 *** (2069)	Non-GM
	GE	−6162 *** (1057)	−16,025 *** (2778)	

Note: ** and *** indicate 10%, 5%, and 1% significance levels, respectively and values in parenthesis are standard errors.

**Table 9:** Comparing WTP with the actual market price for a domestic non-GM product.

		Soybean Oil (900 mL)			Cotton t-Shirt (A Specific SPA Brand)		
		GM (Imported)	Non-GM (Korea)	Organic (Imported)	GM (Imported)	Non-GM (Korea)	Organic (Imported)
WTP	Choice-based conjoint analysis	-	17,704 won	-	-	32,291 won	-
			4.4 times			3.2 times	
	CVM	-	5500 won	-	-	14,500 won	-
			1.4 times			1.5 times	
Market price		4000 won (base)	32,000 won ^1^	11,000 won ^2^	10,000 won (base)	-	20,000 won ^3^
			8 times	2.8 times			2 times

Note: ^1^ Referred to the soybean oil price of the commercial brand Whole Food Story. ^2^ The average soybean oil price of commercial brands Ranieri (12,000 won) and Green Village (10,000 won). ^3^ Based on a cotton t-shirt price of the commercial brand MUJI.

**Table 10:** MWTP variation range for the production technology according to respondents’ characteristics.

Product	Production Technology	MWTP Variation Range (Won)				Reference
		Scientific Knowledge Level ^1^	Sensitivity to Food Safety ^2^	Sensitivity to Technology Safety ^3^	Children’s Age ^4^	
Soybean oil	GM	7594	3762	13,519	11,452	Non-GM
	GE	1036	1645	6990	6131	
Cotton t-shirt	GM	43,909	15,827	-	-	
	GE	40,281	8711	-	-	

Note: Regarding a cotton t-shirt, there were no statistically significant differences in MWTP according to the level difference in sensitivity to technology safety and a children’s age, so they were excluded. ^1^ Divided into four levels (very high, high, low, and very low) based on whether the respondent answered two questions related to GM technology correctly. ^2^ Divided into two levels (high and low) based on whether the respondent purchases food made from organic raw materials. ^3^ Divided into four levels (both are safe, GM is safer, GE is safer, and both are unsafe), based on their perception of the technology safety. ^4^ Divided into three levels: 6 years old or younger, 7–18 years old, and others (unmarried, no children, 19 years old or older).

## Data Availability

The questionnaire presented in this study is available on request from the corresponding author.

## References

[1] American Association for the Advancement of Science (2012). Statement by the AAAS Board of Directors on Labeling of Genetically Modified Foods. https://www.aaas.org/news/statement-aaas-board-directors-labeling-genetically-modified-foods.

[2] Shew A.M., Danforth D.M., Nalley L.L., Nayga R.M., Tsiboe F., Dixon B.L. (2017). New Innovations in Agricultural Biotech: Consumer Acceptance of Topical RNAi in Rice Production. Food Control..

[3] Shew A.M., Nalley L.L., Snell H.A., Nayga R.M., Dixon B.L. (2018). CRISPR Versus GMOs: Public Acceptance and Valuation. Glob. Food Sec..

[4] Korea Biosafety Clearing House (2019). 2018 Major Statistics for Living Modified Organisms.

[5] Ishii T., Araki M. (2016). Consumer Acceptance of Food Crops Developed by Genome Editing. Plant Cell Rep..

[6] Christoph I.B., Roosen J., Bruhn M. Willingness to Pay for Genetically Modified Food and Non-Food Products. Proceedings of the American Agricultural Economics Association Annual Meeting.

[7] Berning J., Campbell B. Consumer Preference and Market Simulations of Food and Non-Food GMO Introductions. Proceedings of the Southern Agricultural Economics Association 2017 Annual Meeting.

[8] Rousu M., Hffian W.E., Shogren J.F., Tegene A. (2003). Should the United States Regulate Mandatory Labeling for Genetically Modified Foods? Evidence from Experimental Auctions. Economics Working Papers.

[9] Buhr B.L., Hayes D.J., Shogren J.F., Kliebenstein J.B. (1995). Valuing Ambiguity: The Case of Genetically Engineered Growth Enhancers. J. Agric. Resour. Econ..

[10] Boccaletti S., Moro D. (2000). Consumer Willingness-to-Pay for GM Food Products in Italy. Agrofórum.

[11] Baker G.A., Burnham T.A. (2001). Consumer Response to Genetically Modified Foods: Market Segment Analysis and Implications for Producers and Policy Makers. J. Agric. Resour. Econ..

[12] Chern W.S., Rickertsen K., Tsuboi N., Fu T. (2003). Consumer Acceptance and Willingness to Pay for Genetically Modified Vegetable Oil and Salmon: A Multiple-Country Assessment. Ag. BioForum..

[13] Kwon O.S. (2003). Estimating the Willingness to Pay for the Non-GMO Agricultural Products: A Contingent Valuation Study. Korean J. Agric. Econ..

[14] Kwon O.S., Kim G.C. (2003). Valuing GMO and Non-GMO Agricultural Products and Experimental Auction Markets. Korean J. Agric. Econ..

[15] Li Q., Curtis K.R., McCluskey J.J., Wahl T.I. (2003). Consumer Attitudes toward Genetically Modified Foods in Beijing, China. AgBioForum.

[16] Lusk J.L. (2003). Effect of Cheap Talk on Consumer Willingness-to-Pay for Golden Rice. Am. J. Agric. Econ..

[17] Lusk J.L., Fox J.A. (2003). Value Elicitation in Retail and Laboratory Environments. Econ. Lett..

[18] Jin J. (2014). Consumer Acceptance and Willingness to Pay for Genetically Modified Rice in China: A Double Bounded Dichotomous Choice Contingent Valuation Survey Calibrated by Cheap Talk. Ph.D. Thesis.

[19] Delmond A.R., McCluskey J.J., Yormirzoev M., Rogova M.A. (2018). Russian Consumer Willingness to Pay for Genetically Modified Food. Food Policy.

[20] Martinez-Ribaya B., Areal F.J. (2020). Is There an Opportunity for Product Differentiation between GM and non-GM Soya-Based Products in Argentina?. Food Control..

[21] Lusk J.L., House L.O., Valli C., Jaeger S.R., Moore M., Morrow B., Traill W.B. (2004). Effect of Information About Benefits of Biotechnology on Consumer Acceptance of Genetically Modified Food: Evidence from Experimental Auctions in the United States, England, and France. Eur. Rev. Agric. Econ..

[22] Lusk J.L., Jamal M., Kurlander L., Roucan M., Taulman L. (2005). A Meta-Analysis of Genetically Modified Food Valuation Studies. J. Agric. Resour. Econ..

[23] Ciriacy-Wantrup S.V. (1947). Capital Returns from Soil-Conservation Practices. J. Farm Econ..

[24] Green P.E., Srinivasan V. (1978). Conjoint Analysis in Consumer Research: Issues and Outlook. J. Con. Res..

[25] Bohm P. (1972). Estimating Demand for Public Goods: An Experiment. Eur. Econ. Rev..

[26] Lancaster K.J. (1966). A New Approach to Consumer Theory. J. Pol. Econ..

[27] McFadden D. (1986). The Choice Theory Approach to Market Research. Mark. Sci..

[28] Voca Moran F.F. (2014). Application of Choice-Based Conjoint Analysis to Determine Consumers’ Preferences and Willingness to Pay for Grass Fed Beef in The United States. Ph.D. Thesis.

[29] Rao V.R. (2014). Applied Conjoint Analysis.

[30] Gujarati D.N., Porter D.C., Park W.G., Hong S.P. (2009). Basic Econometrics of Gujarati.

[31] Vermeulen B., Goos P., Vandebroek M. (2008). Models and Optimal Designs for Conjoint Choice Experiments Including a No-Choice Option. Int. J. Res. Mark..

[32] Hausman J., McFadden D. (1984). Specification Tests for the Multinomial Logit Model. Econometrica.

[33] Eggers F., Sattler H., Teichert T., Völckner F., Homburg C., Klarmann M., Vomberg A. (2018). Choice-Based Conjoint Analysis.

[34] Navrud S., Bråten K.G. (2007). Consumers’ Preferences for Green and Brown Electricity: A Choice Modeling Approach. Rev. Econ. Politiqe.

[35] McFadden D., Zarembka P. (1974). Conditional Logit Analysis of Qualitative Choice Behavior. Frontiers in Econometrics.

[36] Berendsen R. (2015). A Discrete Choice Experiment to Estimate Willingness to Pay for a Microfinance Product in Urban Romania. Master’s Thesis.

[37] Halbrendt C.K., Wirth I.W., Waughn G.F. (1991). Conjoint Analysis of the Mid-Atlantic Food-Fish Market for Farm-Raised Hybrid Striped Bass. South. J. Agric. Econ..

[38] Han J.H., Kim B.S., Joo H.J. (2009). GMO Production and Distribution Status Analysis and GMO Labeling Cost/Benefit Analysis Research.

[39] Ministry of Food and Drug Safety (2017). Labeling Standards for Genetically-Modified Foods, etc..

[40] IDRE Stats-Statistical Consulting Web Resources. https://stats.idre.ucla.edu.

[41] Stanton J., Wirth F.F., Dao Y. (2018). An Analysis of Consumers’ Preferences Between Locally Grown/Processed Food and Organic Food. Curr. Investig. Agric. Curr. Res..

[42] Manalo A.B. (1990). Assessing the Importance of Apple Attributes: An Agricultural Application of Conjoint Analysis. Northeast. J. Agric. Resour. Econ..

[43] Hong W.S., Lee J.S., Park D.S. (2012). Local Food Movement and the Development of Food Service Industries. Korean Hosp. Tour. Acad..

[44] Yang S.B. (2014). Analyzing the Relative Value of Food Labelling on Organic and Origin of Tofu. Korean J. Org. Agric..

[45] Yoo C.H. (2007). EU, Overview of Agricultural Product Quality Policy and Geographic Labeling System.

[46] Miles S., Hafner C., Bolhaar S., Mancebo E.G., Fernández-Rivas M., Knulst A., Hoffmann-Sommergruber K. (2006). Attitudes Towards Genetically Modified Food with a Specific Consumer Benefit in Food Allergic Consumers and Non-Food Allergic Consumers. J. Risk Res..

[47] Tenbült P., de Vries N.K., Dreezens E., Martijn C. (2005). Perceived Naturalness and Acceptance of Genetically Modified Food. Appetite.

